# Management Practices to Build Evidence-Based Decision-Making Capacity for Chronic Disease Prevention in Georgia: A Case Study

**DOI:** 10.5888/pcd15.170482

**Published:** 2018-07-12

**Authors:** Peg Allen, Jean C. O’Connor, Leslie A. Best, Meenakshi Lakshman, Rebekah R. Jacob, Ross C. Brownson

**Affiliations:** 1 Prevention Research Center, Brown School, Washington University in St. Louis, St. Louis, Missouri; 2 Chronic Disease Prevention Section, Georgia Department of Public Health, Atlanta, Georgia; 3 National Association of Chronic Disease Directors, Atlanta, Georgia; 4 The Task Force for Global Health, Decatur, Georgia; 5 Department of Surgery (Division of Public Health Sciences), Alvin J. Siteman Cancer Center, Washington University School of Medicine, Washington University in St. Louis, St. Louis, Missouri

## Abstract

**Background:**

Research shows that training can improve skills needed for evidence-based decision making, but less is known about instituting organizational supports to build capacity for evidence-based chronic disease prevention.

**Community Context:**

The objectives of this case study were to assess facilitators and challenges of applying management practices to support evidence-based decision making in chronic disease prevention programs in the public health system in Georgia through key informant interviews and quantitatively test for changes in perceived management practices and skills through a pre–post survey.

**Methods:**

Leadership of the chronic disease prevention section hosted a multiday training, provided regular supplemental training, restructured the section and staff meetings, led and oversaw technical assistance with partners, instituted transparent performance-based contracting, and made other changes. A 65-item online survey measured perceived importance of skills and the availability of skilled staff, organizational supports, and use of research evidence at baseline (2014) and in 2016 (after training). A structured interview guide asked about management practices, context, internal and external facilitators and barriers, and recommendations.

**Capacity-Building Activities and Survey Findings:**

Seventy-four staff members and partners completed both surveys (70.5% response). Eleven participants also completed a 1-hour telephone interview. Interview participants deemed leadership support and implementation of multiple concurrent management practices key facilitators to increase capacity. Main challenges included competing priorities, lack of political will, and receipt of requests counter to evidence-based approaches. At posttest, health department staff had significantly reduced gaps in skills overall (10-item sum) and in 4 of 10 individual skills, and increased use of research evidence to justify interventions. Use of research evidence for evaluation, but not skills, increased among partners.

**Interpretation:**

The commitment of leaders with authority to establish multiple management practices to help staff members learn and apply evidence-based decision-making processes is key to increased use of evidence-based chronic disease prevention to improve population health.

## Background

Evidence-based decision making for chronic disease prevention (EBDM) involves complex processes, including applying program planning and quality improvement frameworks; engaging partners in assessment and decision making; adapting and implementing evidence-based policies and programs; conducting sound evaluation; and using evaluation findings to improve implementation and reach (1,2). Both individual skills and organizational supports are needed to apply and sustain EBDM processes. A literature review identified 5 domains of management practices with evidence of improving agency performance: leadership support, workforce development, organizational climate and culture, relationships and partnerships, and financial practices (3).

The Centers for Disease Control and Prevention (CDC), along with other funders, expect state public health departments to implement or contract for evidence-based approaches (4). Training in EBDM can improve skills needed to use EBDM processes (5). EBDM is also recognized as having an impact on the effectiveness of chronic disease units (6). But less is known about how to foster organizational climates that support evidence-based practice.

## Community Context

The Chronic Disease Prevention Section (CDPS) of the Georgia Department of Public Health (GDPH) has about 50 staff members that help communities create policy, systems, and environmental changes to reduce risk factors for cardiovascular diseases, cancers, diabetes, asthma, and other chronic conditions, promote the healthy development of children and adolescents, and provide access to early detection and management of chronic conditions. CDPS also funds part of the cost of district health chronic disease coordinators in each of the 18 public health districts.

**Figure 1:**
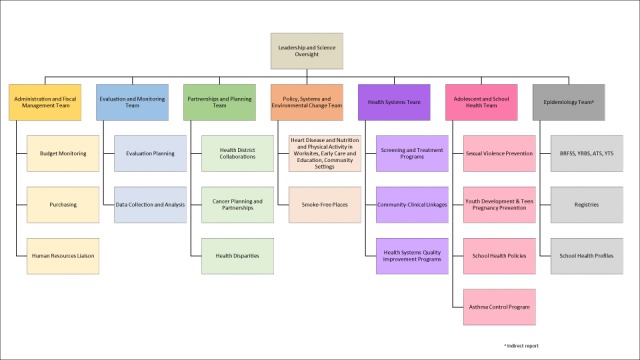
Organizational framework of the Chronic Disease Prevention Section, Georgia Department of Public Health, 2017. Abbreviations: BRFSS, Behavioral Risk Factor Surveillance System; YRBS, Youth Risk Behavior Survey; ATS, Adult Tobacco Survey; YTS, Youth Tobacco Survey.

In fall 2013, a new chronic disease director was hired to move the section toward a coordinated chronic disease approach. To foster collaboration and information sharing to implement evidence-based approaches to reduce risk factors common across disease areas, CDPS was organized into the 4 domains of chronic disease prevention instead of disease area or funding source: epidemiology, environmental approaches, health care systems interventions, and community–clinical linkages (Figure 1). In addition, CDPS focused on the support functions necessary to support EBDM: evaluation and monitoring, administration and fiscal management, and partnerships and planning.

In 2014, recognizing the importance of EBDM, in addition to structure, in achieving improved organizational effectiveness, CDPS also enrolled in a study to assess the impact of an EBDM training and related intervention activities (7,8). The objectives of this case study were to assess facilitators and challenges of applying management practices to support EBDM in the chronic disease prevention programs in the public health system in Georgia through key informant interviews and quantitatively test for changes in perceived management practices and EBDM skills through a pre–post survey.

## Methods

**Figure 2:**
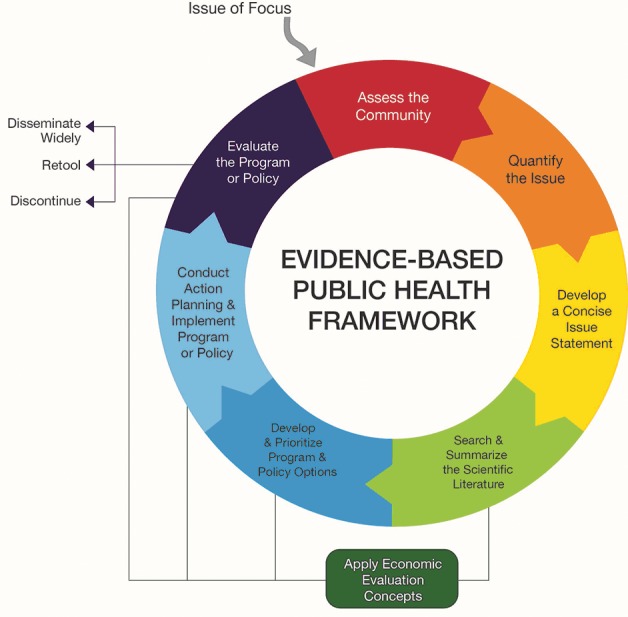
Framework for training in public health evidence-based decision making. Source: Brownson et al (1).

**Participants and data collection.** In August 2014, 30 staff members from CDPS and 1 university partner attended a multiday EBDM training provided by the senior author (R.C.B.) and other faculty members and coordinated by 2 coauthors (R.R.J. and P.A). The content covered 8 modules (Figure 2), plus a ninth introductory module. Time spent on each topic varied from 90 minutes to 3 hours. Attendees participated in interactive lectures, small group exercises, and discussion. As part of a larger study (7,8), a baseline survey was conducted in June and July 2014 (before training) among all 30 professional staff members from CDPS and a purposive sample of 44 partners from other GDPH sections, district and local public health offices, universities, voluntary health agencies, community-based organizations, and other state agencies. Of 124 invited by email, 105 completed the baseline survey (84.7% response). Partners that work closely with, and are sometimes funded by, the programs were included in the survey even though they did not attend the multiday training. The CDPS had implemented management practices to ensure that funded partners were expected or required to perform the contracted work by using an EBDM approach. After the baseline survey was administered, CDPS managers discussed the survey results and lessons learned in the training and collaboratively identified a short list of next steps or problems to be solved to advance EBDM capacity and application. Of 105 participants in the baseline survey, 74 (70.5%) completed the post-training survey in April and May 2016. Five CDPS staff members and 6 partners recommended by the chronic disease director also completed 1-hour structured telephone interviews in 2016.

**Measures**. The 65-item online survey included questions on demographics, the perceived importance of each of 10 EBDM skills (scored on an 11-point Likert scale from 0 = unimportant to 10 = very important) and the availability of agency staff members who have that skill (scored on an 11-point Likert scale from 0 = not available to 10 = very available), frequency of use of research evidence (scored on a 4-point Likert scale from 1 = seldom or never, 2 = sometimes, 3 = often, 4 = always), work unit and agency expectations and supports for EBDM (scored on a 7-point Likert scale from 1 = strongly disagree to 7 = strongly agree), and steps taken to enhance EBDM capacity (check all that apply). The measures were developed from a literature review (3) and the study team’s previous research, and validated through 5 rounds with an expert panel, cognitive response testing with 10 former chronic disease directors, and test–retest reliability with 75 state health department chronic disease staff from other states. The study framework and measures are detailed elsewhere (7,8). Structured interviews asked participants to describe management practices to support EBDM capacity and use, discuss internal and external facilitators and challenges, and recommend ways to enhance and sustain EBDM capacity and application.

**Analyses.** The study team conducted quantitative data management and analyses in SPSS version 24 (IBM Corp). Skill gaps for each of 10 EBDM competencies were calculated as the score for the perceived importance of the skill minus the score for the perceived availability of resources to apply the skill. The sum of the 10 skill gap scores comprised the overall EBDM skill gap. Inferential analyses included paired *t* tests and McNemar tests. Two study team members (P.A., M.L.) independently coded each verbatim interview transcript in NVivo version 10 (QSR International Pty Ltd) and then met to reach consensus. Coded texts were reviewed to identify themes and illustrative quotes.

## Capacity-Building Activities and Survey Findings

**Participant characteristics.** Of the 74 survey participants that completed both baseline and post-training surveys, 30 were from CDPS. The remaining 44 surveys were completed by partners from district, local, and other state governmental agencies (38.6%), coalitions or community-based organizations (34.1%), universities (20.5%), or other organizations (6.8%). Among all participants, most were female (75.7%) and had a master’s or doctoral degree in any field (81.9%). More than one-third (37.5%) had graduate degrees in public health. Nearly half (48.6%) were at least 50 years old. Participant characteristics were similar between CDPS staff members and partners, except partners on average reported more years worked at their agency (median = 10.0 y; interquartile range [IQR], 6.5–13.5 y) and in public health (median = 16.5 y; IQR, 12.5–25.0 y) than CDPS staff members (agency, median = 5.0 y; IQR, 3.0–15.5 y; public health, median = 12.5 y; IQR, 5.0–21.5 y). The 11 participants interviewed (5 CDPS and 6 partners) had worked a median of 15.5 years (IQR, 8.0–25.0 y) in public health and a median of 5.5 years (IQR, 1.0–12.0) in their agencies.

**Capacity-building activities.** Initial steps after the August 2014 EBDM training included revising the staff meeting agendas to incorporate EBDM information with sharing of information across program areas; establishment of a webinar training series for staff and partners, called “Chronic Disease University,” to increase the awareness of the evidence-based strategies for chronic diseases; identifying a mechanism for staff members to have access to the scientific literature; a plan to ensure contracts funded evidence-based strategies and practices; the implementation of management support of opportunities for staff members to publish and present their work; and the addition of expectations to use EBDM approaches into CDPS staff performance plans (Table 1). Three coauthors (R.C.B., R.R.J., P.A.) provided encouragement and guidance through regular conference calls, and 3 coauthors (L.A.B., R.R.J., P.A.) reviewed program overviews. CDPS used the reviews to revise programs and communicate program plans to partners. In spring 2015, the study team provided a 2-session supplemental training on identifying and using publicly available data sources.

**Table 1:** Timeline of Steps to Support and Assess Evidence-Based Chronic Disease Prevention in Georgia, 2013–2017

Date	Chronic Disease Prevention Section Activity	PRC-St. Louis EBDM Training, Support, and Assessment
Fall 2013	CDPS director hired to move CDPS toward coordinated chronic disease approach	—
Spring 2014	CDPS reorganization launched to promote coordinated approach	CDPS enrolled into EBDM study
June–July 2014	—	Baseline pretraining survey of CDPS staff members and partners conducted by R.R.J.
July 2014	Strategic Direction for Chronic Disease Prevention: 2014–2019 published by GDPH	—
August 18–21, 2014	EBDM 3½-day training provided in Atlanta with CDPS staff and GDPH epidemiologists	August 2014 EBDM training provided by R.C.B. and other course faculty
September 2014	EBDM training attendees provided input for next steps selected by CDPS management team	PRC-St. Louis–provided Qualtrics survey for prioritization input on steps brainstormed at August 2014 training
October 2014	Staff meetings reorganized to incorporate EBDM and sharing of information across programs	Monthly collaborative calls of PRC-St. Louis and CDPS started for encouragement and support
November 2014	Statewide Chronic Disease Council advisory body of 24 leaders from diverse sectors launched by CDPS	—
November 2014 and January 2015	Summary presentations created by staff in each CDPS program for program review, revision, and communication to partners	Review of programs by L.A.B., P.A., R.R.J.
January 2015	CDPS commitment to follow science and EBDM processes, including incorporation of EBDM in CDPS manager and program staff annual performance plans	—
January–May 2015	20 new CDPS staff members, many with MPH degrees and PhDs, hired and brought on board	—
May 2015	CDPS annual meeting held with local health district chronic disease managers	—
May 2015 to Dec 2016	Statewide health assessment and health improvement plan led by CDPS as part of GDPH’s accreditation preparations	Using Data Sources for Public Health Practice supplementary 2-session webinar training provided to CDPS, other GDPH staff
June 2015	CDPS website relaunch completed, with posting of chronic disease data and program information, logic models, evaluation plans and reports, and links to resources to enhance partner access to information for evidence-based public health practice	—
August 2015	STAR site visit with the National Association of Chronic Disease Directors took place, with feedback to identify strengths and ways CDPS could improve	—
Fall 2015	A plan for how to institute STAR recommendations developed by CDPS leadership team	—
July 2015	Chronic Disease University monthly webinar series launched with J.C.O.’s EBDM overview	R.C.B. and P.A. contributed slides to J.C.O.’s EBDM introductory overview to the series
April–May 2016	—	Post-training survey of CDPS staff and partners conducted by P.A. and M.L.
May 2016	CDPS annual meeting held with local health district chronic disease managers	—
May–June 2016	—	11 post-training interviews conducted by L.A.B. and P.A.
August 2016	Retreat held by CDPS leadership team to identify continuing implementation actions	—
Winter 2016–Spring 2017	CDPS participated in a STAR follow-up site visit and identified follow-up items, including staff survey; CDPS facilitated GDPH enrollment in the Public Health Digital Library, providing all staff with journal access via the GDPH intranet	Quantitative data management, analyses by P.A.; qualitative coding, analyses by P.A., M.L.
May 2017	CDPS annual meeting with local health district chronic disease managers; CDPS staff member wins the departmental award for excellence in science	—
September 2017	All staff surveyed on organizational culture and opportunities for improvement; CDPS held an all-staff strategic planning retreat to evaluate progress; CDPS leadership team held a retreat to identify continuing implementation actions	—

Abbreviations: CDPS, Chronic Disease Prevention Section; EBDM, evidence-based decision making; GDPH, Georgia Department of Public Health; PRC-St. Louis, Prevention Research Center in St. Louis, Brown School, Washington University in St. Louis; STAR, State Technical Review and Assistance Program provided by the National Association of Chronic Disease Directors on behalf of Centers for Disease Control and Prevention.

Concurrently, the CDPS director and her leadership team revised the section’s strategic plan in 2014; convened an advisory council with university, business, health care, and public health leaders in November 2014 that meets quarterly; reformatted staff meetings to increase cross-program sharing; prepared for a fall 2015 review by the National Association of Chronic Disease Directors that assessed core components of a successful section and provided positive feedback; developed a leadership plan from the recommendations; sought and obtained additional guidance from several CDC employees; expanded university partnerships; and supported staff in presenting at state and national conferences.

Interviews of CDPS staff members resulted in quotes that describe the EBDM capacity-building management practices instituted by the CDPS director and her leadership team (Table 2). Several participants emphasized the importance of multiple management practices and some of the structural changes in how programs were organized, stating that any single approach would not have institutionalized EBDM. Partners received several supports from CDPS: menus of evidence-based approaches in requests for local proposals, evidence information, technical assistance, training, and review of work plans. District health promotion coordinators attended annual in-person updates and monthly webinars, and CDPS posted webinar slides online. Examples of technical assistance provided by CDPS staff included helping with implementation plans and developing measures and surveys. Chronic disease indicators and resources were posted online. Partners appreciated the public access to GDPH’s central data repository and provision of additional data on request. Local coalitions received trainings from CDPS or contractors to help develop coalitions and prepare for evidence-based coalition-driven strategies. Two of 5 CDPS interview participants deemed partner training and development, and review of partner plans and progress, as the most successful aspects of CDPS’ promotion of EBDM, while the others considered leadership support as most influential.

**Table 2:** Descriptions of Management Practices Instituted to Support Evidence-Based Decision Making Provided by 5 Interviews of CDPS Staff Members, 2016

Domain	Management Practice	Sample of Quotes That Describe the Management Practice	Additional Comments
Leadership support in CDPS	Role modeled EBDM	Bringing together the art and science has raised our awareness of how evidence contributes to strengthening our programs and our collective work and that is something very different that the chronic disease director has done.	—
	Emphasized and expected EBDM	I think EBDM started with the expectation that it was part of your job, your job description, and part of your goals that you needed to meet every year. I think that was probably the most effective way to support EBDM.	Expectations communicated through repetition both verbally and through an internal review process of program plans.
	Supported and protected staff	And protect them and support them when they are doing the right thing, but are being given a hard time by other people . . . addressing the safety issues for people about making some of these changes.	—
	Incorporated EBDM in staff meetings	I would often see staff in meetings with staff from a different unit, talking about a program or initiative, and that was the biggest change, seeing that interaction . . . . working together on an initiative or an evaluation.	—
	Provided supportive tools	Providing “monthly webinars” [with posted slides], “making fact sheets and putting it up on our website, providing them [partners] with those materials.	Posted chronic disease indicators and data source descriptions on section webpages. Collaborated with a university library to increase access to full-text journal articles.
Restructuring of CDPS	Restructured section by function	It has forced programs and forced people who have similar risk factors to deal with from a disease category to work together . . . so our current configuration has broken the silos.	—
	Restructured programs	We have completely redone some of our programs.	Restructured programs to prioritize evidence-based policy, environmental, and systems approaches.
	Restructured program planning	Shifted how we do program planning here . . . now the first question is what does the Community Guide say . . . the literature.	Changed program planning processes to be evidence-driven and assigned evaluators to help with planning.
Workforce development	Hiring qualified staff	We are hiring, everyone has at least a master’s degree or an MPH. And people are coming with a lot of experience.	—
	Job descriptions	All staff who have a programmatic job do have a science component to their job description . . . a requirement that they use the literature, the evidence, know how to cite and refer to those kinds of sources, and apply them in their work.	—
	Performance reviews	Performance management plan for the year . . . requires them to present or submit abstracts, for example, to conferences and so helps to promote the use of evidence-based processes . . . more than 20 abstracts were accepted and presented at different conferences.	—
	Chronic Disease Webinar Series	To showcase their work to other staff and external partners . . . a time for staff to talk about a facet of evidence-based public health and how it impacts their program.	https://dph.georgia.gov/chronic-disease-university
	New employee orientation to EBDM	We made sure that all the staff that came on board after that training [EBDM] were exposed or given the opportunity to go and train.	EBDM course slides posted on staff intranet. Trained staff discussed course and shared materials, and a few new staff attended the national Brownson EBDM course.
	External training opportunities	That plan [annual employee performance management plan] also, for most of our programmatic staff, requires to present or submit abstracts, for example, to conferences, and so helps to promote the use of EBDM processes.	Staff encouraged to attend external trainings. If staff present at a conference, they can then attend the full conference as part of their continued learning.
	Evaluation training series	To explain what evaluation is and how it should be married to program development and how we should be evaluating in our partnerships.	Program logic models, evaluation plans posted as well.
Organizational climate	Acceptance of EBDM expectations	[After initial mixed views], the culture was that people accepted it. They understood it was something we all had heard about in grad school, we had been introduced to it, but now it was time to practice it.	—
	A pull to and away from EBDM	We do have a little bit of tension in that our CDC-funded work really requires evidence-based approaches . . . and then on the other hand we get requests . . .that aren’t evidence-based.	—
Relationships and partnerships	Participatory decision making	We present them [funded partners] with a list of options . . . so we have discussions about that . . . and it can go back and forth for some time . . .we try not to dictate what it is that they have to do.	—
Financial practices	Performance-based contracting	For any contract that we’re going to put out there for a program . . . the outcomes and objectives have to be based in the evidence.	—
	Transparency	[CDPS] has made a great effort to be as transparent as [CDPS director] can be with budget issues, with programming issues, and I really appreciate that . . . as transparent as possible with the requests for proposals, the timelines, the timeframes that we need to get things done.	—

Abbreviations: CDC, Centers for Disease Control and Prevention; CDPS, Chronic Disease Prevention Section of the Georgia Department of Public Health; EBDM, evidence-based decision making.

Partners also supported CDPS by carrying out the evidence-based interventions or promoting them with other local entities, sharing success stories, modeling success for others, lending expertise to coalitions, providing training and technical assistance with local partners, and maintaining linkages with nearby universities. Some partners, especially university-based partners, “actually were a part of that call” for strengthening EBDM in Georgia and have been supportive since “they wanted the state programs to function” with more impact. Participants discussed EBDM partnering successes, including increased coalition capacity to implement policy changes, credibility, and ability to get grants, networking skills, reach, and value of evaluation.

**Facilitators**. Leadership support of the chronic disease director was cited repeatedly by GDPH staff and partners as the key facilitator to EBDM capacity building (Table 2). Additional facilitators discussed by GDPH staff included consistent messaging on EBDM internally and with partners; the section reorganization from a disease specific to a coordinated approach to chronic disease prevention; and evaluation staff working alongside program staff “so we know what’s working and not working and how to plan.” In addition to CDPS leadership support, partners emphasized CDPS technical assistance, relationships with local partners, and increased availability of online data from GDPH (Table 2). Partners also offered the following as facilitators: request for proposal requirements to use EBDM where “you have to have your ducks in a row beforehand”; having to report progress to GDPH or other funders; CDPS transparency in posting program budgets online and notice of funding opportunities for local public health districts; having a nearby university partner; aligning with community priorities; district health department accreditation processes where “everything is EBDM because there has to be a justification for everything that we do;” and “more frequent,” “more structured,” and “open” communication with CDPS. To participants, EBDM was not just a term, “it is in everything that we do now . . . it’s living and breathing and in my face all of the time, which is great.”

**Challenges**. Both partners and CDPS staff stated that competing local priorities, varied local or district leadership support, social norms, or local politics sometimes meant chronic disease prevention takes a back seat. A lack of state or local political will “sometimes means that we can’t implement what’s evidence-based practice,” depending on the “needs and appetites of the political machine*.*” Sometimes “we get requests that aren’t evidence-based.” Additional common challenges were time and varied EBDM familiarity and skill levels.

CDPS staff also mentioned hiring restrictions, inability to offer competitive salaries, fear of conflict with partners, resistance to change, lack of clarity from funders, societal lack of belief in prevention, a lack of alignment between partner organization goals and funding requirements, and difficulty finding partners willing to work across racial and economic lines. Partners discussed additional challenges of not having enough staff to address chronic disease prevention in the many counties each district serves, lack of public health training in local health departments, some partners not seeing where they fit into EBDM processes, lack of lead time to develop local coalitions for requests for proposals, lack of local grant writers, cultural and language barriers, little understanding among newer GDPH staff members about what districts can and cannot do, conflicts between county and state agendas, and lack of “artful” communication of evidence “in a way to get conversation going.” One interviewee said, “Folks have not always agreed that the evidence is right, especially if it goes against some held belief or value, and so that’s always a challenge.” Another one said, “And there’s also the politics of race and economics. A fair amount of that is divided along urban and rural lines, but it’s a real challenge.”

**Quantitative findings**. Among CDPS staff members, the largest EBDM skill gaps at baseline were in prioritization, adapting interventions, and understanding how to use economic evaluation information (Table 3). Mean perceived skill gaps significantly decreased overall (*P* = .02), in prioritization (*P* = .01), adapting interventions (*P* = .005), qualitative evaluation (*P* = .04), and action planning (*P* = .01). Mean perceived supervisory expectations for use of EBDM increased (*P* = .006), as did perceived work-unit access to staff resources for EBDM (*P* = .01). Use of research evidence to justify selection of interventions increased (*P* = .008). Among the 44 partners, use of research evidence to evaluate interventions increased (*P* = .03). 

**Table 3:** Changes From Baseline to Post-Training in Skill Gaps in Evidence-Based Decision Making, Use of Research Evidence, and Organizational Supports, CDPS Staff Members (n = 30) and Staff Members From Partnering Organizations (n = 44), 2014–2016^a^

Survey Item	CDPS Staff (n = 30)			Partners^b^ (n = 44)		
	Baseline Mean (SD)	Post-Training Mean (SD)	*P* Value for Paired *t* Test	Baseline Mean (SD)	Post-Training Mean (SD)	*P* Value for Paired *t* Test
**Work-unit skill gaps^c^**						
Prioritization	2.2 (2.4)	1.0 (1.2)	.01	1.2 (2.0)	0.9 (1.3)	.41
Adapting interventions	2.4 (2.3)	1.1 (1.9)	.005	1.4 (2.4)	1.4 (2.4)	.99
Quantifying the issue	1.1 (2.1)	1.1 (2.3)	.94	0.9 (2.2)	0.8 (2.2)	.82
Evaluation designs	1.6 (2.4)	0.9 (1.7)	.23	1.7 (2.2)	1.0 (2.0)	.13
Quantitative evaluation	1.3 (1.8)	0.7 (1.9)	.10	1.2 (1.8)	1.3 (1.9)	.69
Qualitative evaluation	1.8 (2.6)	0.7 (1.9)	.04	1.6 (2.7)	1.4 (2.1)	.74
Economic evaluation	3.3 (3.4)	3.2 (2.8)	.96	2.0 (2.2)	2.3 (3.0)	.56
Action planning	1.5 (1.9)	0.6 (1.0)	.01	0.9 (1.5)	0.9 (1.4)	.95
Community assessment	1.7 (1.6)	1.1 (1.2)	.15	1.0 (1.6)	1.6 (2.3)	.16
Communicating research to policymakers	1.9 (2.6)	1.6 (2.1)	.57	1.7 (2.0)	1.7 (2.3)	.87
Overall (10-item sum)	19.0 (17.1)	11.7 (11.9)	.02	13.8 (15.7)	13.5 (15.0)	.94
**Use of research evidence for job tasks^d^**						
Write a grant application	2.7 (0.6)	2.8 (0.5)	.77	2.6 (0.6)	2.7 (0.5)	.79
Plan or conduct a needs assessment	2.6 (0.6)	2.5 (0.6)	.34	2.8 (0.4)	2.6 (0.6)	.14
Select an intervention	2.6 (0.6)	2.7 (0.4)	.20	2.8 (0.4)	2.8 (0.4)	.71
Justify intervention selection to funders and leadership	2.4 (0.8)	2.9 (0.2)	.002	2.9 (0.4)	2.7 (0.5)	.29
Evaluate interventions	2.7 (0.6)	2.6 (0.7)	.54	2.8 (0.4)	2.6 (0.5)	.03
Develop materials for partners	2.5 (0.7)	2.7 (0.5)	.21	2.8 (0.4)	2.8 (0.4)	.53
**Organizational supports^e^**						
My direct supervisor expects me to use EBDM	5.4 (1.4)	6.1 (1.1)	.006	5.5 (1.4)	5.4 (1.4)	.74
My direct supervisor recognizes the value of management practices that facilitate EBDM	5.5 (1.5)	5.9 (1.2)	.04	5.8 (1.2)	5.3 (1.3)	.01
My performance is partially evaluated on how well I use EBDM in my work	4.1 (1.7)	4.3 (1.7)	.52	4.9 (1.4)	4.4 (1.4)	.04
My work unit has access to current research evidence for EBDM	5.1 (1.6)	5.6 (1.4)	.18	5.7 (1.2)	5.8 (1.0)	.71
My work unit has the resources (eg, staff, facilities, partners) to support application of EBDM	4.5 (1.5)	5.2 (1.4)	.01	4.7 (1.7)	4.7 (1.6)	.94
The staff in my work unit has the necessary skills to carry out EBDM	4.9 (1.6)	5.6 (1.2)	.04	5.2 (1.5)	5.2 (1.3)	.99
Information is widely shared in my work unit for decision making	4.9 (2.0)	5.3 (1.6)	.12	5.6 (1.2)	5.6 (1.1)	.91
My work unit distributes intervention evaluation findings to other organizations	5.0 (1.9)	5.6 (1.6)	.09	5.4 (1.4)	5.6 (1.4)	.58
My agency is committed to hiring people with relevant training in core disciplines in public health	5.0 (1.8)	5.7 (1.5)	.01	5.4 (1.6)	5.4 (1.2)	.99

Abbreviation: CDPS, Chronic Disease Prevention Section of the GDPH; GDPH, Georgia Department of Public Health; EBDM, evidence-based decision making.

a Baseline survey was conducted before training in June and July 2014; of 124 potential participants (30 CDPS staff members and 94 partners) invited by email, 105 completed the baseline survey (84.7% response). Of those who completed baseline survey, 74 (70.5%) completed the post-training survey in April and May 2016.

b From other GDPH sections, district and local public health offices, universities, voluntary health agencies, community-based organizations, and other state agencies.

c Calculated as the score for the perceived importance of the skill in the work unit minus the score for the perceived availability of resources for applying the skill in the work unit. Both importance and availability were scored on an 11-point Likert scale (from 0 = not important to 10 = very important and 0 = not available to 10 = very available). The question on the survey was, “Now, we would appreciate your help rating the importance and availability of each skill in the statements below. First, read the statements (skills in EBDM) below; then, use the first scale to rate the importance of each of the skills to you. Next, use the second scale to rate how available each skill is to you when you need it (either in your own skill set or among others in your agency).”

d Participants provided responses on a 4-point Likert scale: 1 = seldom or never, 2 = sometimes, 3 = often, 4 = always.

e Participants provided responses on a 7-point Likert scale: 1 = strongly disagree to 7 = strongly agree.

Commonly reported steps to improve capacity for EBDM, as reported by CDPS and partner survey participants, were informally sharing EBDM knowledge (53%), using EBDM knowledge to plan (43%) or evaluate (33%) a program, and building EBDM principles into employee performance expectations (27%).

**Participant recommendations**. CDPS recommendations focused on training, hiring, and educating decision makers about prevention effectiveness. Training recommendations included 1) a standardized EBDM orientation for new staff, 2) more skills-based training, not just informational trainings, and 3) more training in policy change. Staff wanted planned hiring of people experienced in policy change to continue to increase the section’s capacity to guide partners. And because of lack of belief in prevention in some areas, “it’s convincing people that prevention works. I think we have to get better at doing that.”

Partners made similar recommendations, and also wanted CDPS to 1) provide summaries of emerging issues such as e-cigarettes, 2) pool and distribute information important to communities, 3) increase interagency collaboration at the state level, 4) give as much notice as possible of upcoming proposals to allow time for local partnering in preparation, and 5) involve district staff in drafting requests for proposals. Partners also recommended hiring more district and local health promotion staff so in-person relationship building can lay the groundwork for increased local capacity to implement evidence-based practice.

## Interpretation

This case study describes multiple management strategies used to build EBDM capacity in the chronic disease prevention unit of a state health department in the areas of leadership support, workforce development, partnering, and financial practices. After training and institution of management practices, self-reported EBDM skills and availability of resources for EBDM improved among CDPS staff. Use of research evidence increased among CDPS staff and partners.

Leadership support and the use of multiple management strategies rather than a single strategy emerged as the key facilitators of EBDM capacity building among interview participants. In qualitative studies in Canada, participants deemed leadership role modeling, expectations of EBDM, and provision of staff time to learn and apply EBDM processes instrumental in building EBDM capacity and use (9–11). Aarons and colleagues encourage leaders at all levels to role model and coach EBDM, pay regular attention to EBDM, stand by EBDM principles in stressful situations, direct resources to evidence-based policies and programs, acknowledge staff for applying EBDM processes, and employ and promote those experienced in EBDM or at least open to EBDM (12). Our case study showed that the chronic disease director used these and additional approaches to build capacity.

An additional strategy was the reorganization of CDPS by role instead of chronic condition, with the intent to facilitate cross-program data sharing and collaboration to better plan and implement evidence-based approaches to reduce risk factors such as obesity and tobacco use, which are common to multiple chronic diseases. In a case study of structure, chronic disease prevention staff found that collaboration was enhanced by having specialist groups meet across program areas (6). A public health department in Ontario, Canada, found that forming formal study groups across administrative boundaries facilitated ongoing communication and collaboration across programs (10). Participants in our case study reported that the formal administrative realignment facilitated communication across program areas and that it was too soon to know the full impact of the reorganization.

Workforce development was multifaceted, as recommended by Roche and colleagues (13), with ongoing supplemental webinars after the initial multiday training, internal program review, external technical assistance with partners (and at times provided by other partners), recruitment of employees with a master’s degree in public health, verbal reinforcement of EBDM principles and expectations, and incorporation into employee development plans within the constraints of the state performance review format.

Managing change with communication is critical to building EBDM capacity. Health department staff and public health leaders emphasize the importance of clear communication of EBDM principles, expectations, and processes, and sharing of information across programs (2,9–11,14–17). A health department in Ottawa, Canada, found that focusing solely on obtaining and disseminating evidence was not enough, as staff had to learn to think about different ways of approaching their jobs, which raised anxieties (10). Staff emphasized the importance of strategizing for the complexity of the changes and framing EBDM capacity building as a long-term process to allow adequate time to identify and accomplish realistic individual and organizational objectives. Supporting staff when challenged by others for EBDM processes (eg, a priority change driven by evidence) was a helpful change-management strategy in our case study and in the Ottawa health department (9). Steps taken in our case study were well aligned with recommendations for public health managers to be a change agent, use a structured process to manage change, address support and resistance to change, and recruit staff with skills to sustain change management (16). To address changes from evolving evidence and external political forces, public health change managers need skills in systems thinking, communication, organizational behavior, ethics-based approaches to achieve health equity, and policy assessment and translation (17).

The efforts to have transparent budget processes, requests for proposals, and timelines were appreciated in partner interviews. Partners wanted more lead time for requests for proposals to work through local partnerships in time to apply competitively, but they also expressed understanding of timeline constraints. Performance-based contracts ensure that resources support evidence-based planning, implementation, evaluation, and enhanced transparency in the contracting process (18).

Challenges to EBDM expressed by participants reflected barriers noted in earlier studies (4,19), where managers were recommended to acknowledge that EBDM processes are time consuming, protect staff time for EBDM processes (10,20), and create an organizational climate that promotes a balance of thinking and doing instead of all doing (10).

In addition to recommendations from interview participants, we recommend the following: 1) ongoing development among leadership teams and staff members in skills not only in EBDM but also in technical assistance, communication, strategic planning, policy development, and coalition building; 2) replication of EBDM training with nonuniversity chronic disease prevention partners; 3) long-term commitment to EBDM communication and change management; 4) enhanced engagement in participatory decision making as staff and partner skills in EBDM and communication grow; and 5) addressing health equity challenges. Health departments are directing resources to areas with poor health outcomes but lack of political will for EBDM persists in some corners. Skepticism about the effectiveness of prevention, racism and social inequality, and reluctance in some communities to partner across racial, economic, and rural/urban strata are common challenges requiring nontraditional transdisciplinary work (2).

Our case study has several limitations. Generalizability is limited because of the context of the study. The small sample sizes (30 CDPS staff members, 44 partners) made it difficult to detect significant change. The self-reported Likert items on organizational supports may not fully measure actual management practices.

Leadership support is critical for EBDM capacity building and dissemination. Commitment of leaders within agencies with authority and skills to institute multiple management practices and help staff learn and apply EBDM processes is important for the spread of evidence-based chronic disease prevention and improved population health.

## References

[1] Brownson RC , Baker EA , Deshpande AD , Gillespie KN . Evidence-based public health. Third ed. New York (NY): Oxford University Press; 2018.

[2] Brownson RC , Fielding JE , Green LW . Building capacity for evidence-based public health. Annu Rev Public Health 2018;39:27–53. 10.1146/annurev-publhealth-040617-014746 29166243 PMC5972383

[3] Brownson RC , Allen P , Duggan K , Stamatakis KA , Erwin PC . Fostering more-effective public health by identifying administrative evidence-based practices: a review of the literature. Am J Prev Med 2012;43(3):309–19. 10.1016/j.amepre.2012.06.006 22898125 PMC3990249

[4] Steele CB , Rose JM , Townsend JS , Fonseka J , Richardson LC , Chovnick G . Comprehensive cancer control partners’ use of and attitudes about evidence-based practices. Prev Chronic Dis 2015;12:E113. 10.5888/pcd12.150095 26182148 PMC4509093

[5] Jacob RR , Baker EA , Allen P , Dodson EA , Duggan K , Fields R , Training needs and supports for evidence-based decision making among the public health workforce in the United States. BMC Health Serv Res 2014;14(1):564. 10.1186/s12913-014-0564-7 25398652 PMC4245845

[6] Alongi J . A case study examination of structure and function in a state health department chronic disease unit. Am J Public Health 2015;105(Suppl 2):e15–22. 10.2105/AJPH.2014.302354 25689211 PMC4355693

[7] Allen P , Sequeira S , Jacob RR , Hino AA , Stamatakis KA , Harris JK , Promoting state health department evidence-based cancer and chronic disease prevention: a multi-phase dissemination study with a cluster randomized trial component. Implement Sci 2013;8(1):141. 10.1186/1748-5908-8-141 24330729 PMC3878781

[8] Brownson RC , Allen P , Jacob RR , deRuyter A , Lakshman M , Reis RS , Controlling chronic diseases through evidence-based decision making: a group-randomized trial. Prev Chronic Dis 2017;14:E121. 10.5888/pcd14.170326 29191262 PMC5716810

[9] Ward M , Mowat D . Creating an organizational culture for evidence-informed decision making. Healthc Manage Forum 2012;25(3):146–50. 10.1016/j.hcmf.2012.07.005 23252330

[10] Peirson L , Ciliska D , Dobbins M , Mowat D . Building capacity for evidence informed decision making in public health: a case study of organizational change. BMC Public Health 2012;12(1):137. 10.1186/1471-2458-12-137 22348688 PMC3305606

[11] Zardo P , Collie A , Livingstone C . Organisational factors affecting policy and programme decision making in a public health policy environment. Evid Policy 2015;11(4):509–27. 10.1332/174426414X14170304008766

[12] Aarons GA , Ehrhart MG , Farahnak LR , Sklar M . Aligning leadership across systems and organizations to develop a strategic climate for evidence-based practice implementation. Annu Rev Public Health 2014;35(1):255–74. 10.1146/annurev-publhealth-032013-182447 24641560 PMC4348088

[13] Roche AM , Pidd K , Freeman T . Achieving professional practice change: from training to workforce development. Drug Alcohol Rev 2009;28(5):550–7. 10.1111/j.1465-3362.2009.00111.x 19737213

[14] Hardy AK , Nevin-Woods C , Proud S , Brownson RC . Promoting evidence-based decision making in a local health department, Pueblo City-County, Colorado. Prev Chronic Dis 2015;12:E100. 10.5888/pcd12.140507 26111156 PMC4492218

[15] Winterbauer NL , Bridger CM , Tucker A , Rafferty AP , Luo H . Adoption of evidence-based interventions in local health departments: “1-2-3 Pap NC”. Am J Prev Med 2015;49(2):309–16. 10.1016/j.amepre.2015.02.024 26190805

[16] Thompson JM . Understanding and managing organizational change: implications for public health management. J Public Health Manag Pract 2010;16(2):167–73. 10.1097/PHH.0b013e3181c8cb51 20150801

[17] Erwin PC , Brownson RC . The public health practitioner of the future. Am J Public Health 2017;107(8):1227–32. 10.2105/AJPH.2017.303823 28640683 PMC5508141

[18] Honoré PA , Simoes EJ , Moonesinghe R , Kirbey HC , Renner M . Applying principles for outcomes-based contracting in a public health program. J Public Health Manag Pract 2004;10(5):451–7. 10.1097/00124784-200409000-00013 15552771

[19] Jacobs JA , Dodson EA , Baker EA , Deshpande AD , Brownson RC . Barriers to evidence-based decision making in public health: a national survey of chronic disease practitioners. Public Health Rep 2010;125(5):736–42. 10.1177/003335491012500516 20873290 PMC2925010

[20] Traynor R , Dobbins M , DeCorby K . Challenges of partnership research: insights from a collaborative partnership in evidence-informed public health decision making. Evid Policy 2015;11(1):99–109. 10.1332/174426414X14043807774174

